# Humic acids alleviate dextran sulfate sodium-induced colitis by positively modulating gut microbiota

**DOI:** 10.3389/fmicb.2023.1147110

**Published:** 2023-04-12

**Authors:** Jiazhang Huang, Pengfei Xu, Mingzhi Shao, Bin Wei, Cong Zhang, Jie Zhang

**Affiliations:** ^1^State Key Laboratory of Biobased Material and Green Papermaking, School of Bioengineering, Qilu University of Technology (Shandong Academy of Sciences), Jinan, China; ^2^Institute of Food and Nutrition Development, Ministry of Agriculture and Rural Affairs, Beijing, China; ^3^Ultrasound Department of Zhucheng People's Hospital, Weifang, China; ^4^Shandong Asia-Pacific Haihua Biotechnology Co., Ltd., Jinan, China

**Keywords:** humic substances, gut microbiota, inflammation, traditional Chinese medicine, enteritis

## Abstract

Humic acids (HAs) are natural polymers with diverse functional groups that have been documented and utilized in traditional Chinese medicine. Dextran sulfate sodium (DSS)-induced colitis has been used as a model to study inflammatory bowel disease. In this research, we investigate the effect of HAs on ameliorating DSS-induced colitis in mice. Our aim here was to investigate if HAs could be a remedy against colitis and the mechanisms involved. The results show that HAs facilitated a regain of body weight and restoration of intestinal morphology after DSS-induced colitis. HAs treatment alters the community of gut microbiota with more *Lactobacillus* and *Bifidobacterium*. Changes in bacterial community result in lower amounts of lipopolysaccharides in mouse sera, as well as lower levels of inflammatory cytokines through the Toll-like receptor 4 (TLR4)-NF-κB pathway. HAs also promoted the expression of tight junction proteins, which protect the intestinal barrier from DSS damage. Cell experiments show that HAs display an inhibitory effect on DSS growth as well. These results suggest that HAs can alleviate colitis by regulating intestinal microbiota, reducing inflammation, maintaining mucosal barriers, and inhibiting pathogen growth. Thus, HAs offer great potential for the prevention and treatment of colitis.

## 1. Introduction

Diarrhea and colitis caused by bacterial infection are common diseases in both humans and animals (Hauck et al., 2016; Sun et al., 2022; Jia et al., 2023). Antibiotics have been widely used to treat bacteria-induced colitis. In addition, antibiotics have been exploited to promote animal growth and reduce production costs in livestock breeding. Microbial resistance caused by the misuse of antibiotics has become a serious problem globally (Rivera-Sánchez et al., 2022; Cui et al., 2023). Therefore, it is necessary to explore alternatives to antibiotics for treating colitis.

**Figure 1:**
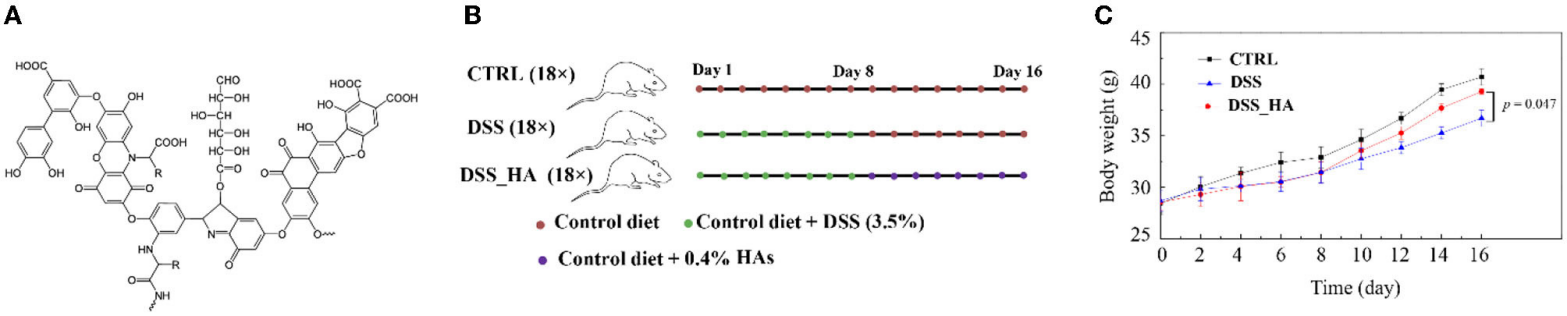
Effect of HAs on colitisin mice. **(A)** Chemical structure of HAs. **(B)** Study design for the three mouse cohorts (*n* = 18 × 3). The three cohorts were fed a control diet (CTRL), a diet containing DSS (DSS), and a diet containing HAs (DSS_HAs) as indicated. **(C)** Body weight gain in mice of the three cohorts.

Humic acids (HAs) are macromolecular organic substances rich in hydroxyl, carboxyl, and other groups (Figure 1A), which are formed from plant and microbial residues through humification (Sarlaki et al., 2019; Xu et al., 2022). In Chinese traditional medicine, HAs have been called “WU-JIN-SAN” (乌金散) since 1786 (Miao et al., 2018). HAs provide molecules with antibacterial, anti-inflammatory, antioxidant, and immunomodulatory functions (Mudronová et al., 2021). Moreover, HAs have been used to treat various diseases, including diarrhea, gastritis, gastric ulcers, and colitis (Rusliandi et al., 2020; Socol, 2023).

Previous studies have shown that adding HAs and lincomycin to the diet can improve the growth performance, nutrient digestibility, blood biochemistry, and intestinal morphology of broiler chickens infected with *Clostridium* (Saleh et al., 2022). Adding 10% HAs can significantly increase the body weight and feed utilization of finishing pigs (Wang et al., 2008). As far as we know, there are no other studies evaluating the effect of HAs on DSS-induced colitis in mice. The purpose of this study was to evaluate the effects of adding HAs on growth performance, intestinal morphology, intestinal flora, and inflammatory pathway of mice exposed to DSS. The results showed that HAs were helpful in the recovery of body weight and intestinal morphology after colitis. HAs can alleviate colitis by regulating intestinal microflora, reducing inflammation, and maintaining the mucosal barrier. Therefore, HAs have great potential in the prevention and treatment of colitis.

## 2. Materials and methods

### 2.1. Ethics statement

All animal experiments followed the protocols approved by the Animal Care and Use Committee of the Qilu University of Technology (Shandong Academy of Sciences).

### 2.2. Animals

Healthy Kunming mice were obtained from Pengyue Laboratory Animal Breeding Co., Ltd. (Jinan, China; experimental animal use license: SCXK (Lu) 20140007). A total of 54 Kunming mice weighing 28 ± 2 g were randomly divided into three treatment groups, with half male and half female mice in each group. The mice were managed with natural ventilation, *ad libitum* feeding, and free access to drinking water.

### 2.3. Experimental design

The CTRL group was administered purified water throughout the whole process; mice in the DSS and DSS_HA groups were fed water containing DSS (3.5%) for 8 days. From day 9, the DSS group was fed distilled water with a normal diet. Mice in the DSS_HA group were fed distilled water containing 0.4% HAs (Shandong Yatai, China) every day (Figure 1B). During the experiment, the body weight was measured every 2 days, and the mental state, diet, water intake, and the final characteristics of the mice were observed and recorded.

### 2.4. Histological staining

Mice were euthanized by cervical dislocation. The duodenum, the jejunum, the ileum, and the colon (1 cm) were selected, washed with 0.9% normal saline, and then fixed in 4% formaldehyde. The fixed intestines were encapsulated and sliced. After staining with hematoxylin and eosin (H&E), the morphology of the tissues was examined using light microscopy.

### 2.5. mRNA and protein level detection

The colon tissue was isolated and homogenized. Total RNA was purified and reversely transcribed using PrimeScript^®^ RT reagent kit with gDNA Eraser (Takara, Dalian, China). SYBR^®^ Premix Ex Taq™ (Takara, Dalian, China) was used for fluorescence quantitative PCR, according to the kit method. The GAPDH gene was used as the internal reference. The primers for TLR4 were as follows: GCTTTCACCTCTGCCTTCAC-3′ and 5′-CGAGGCTTTTCCATCCAATA-3′. The primers for *ZO-1* were as follows: 5′-GAG-GATGGTCACACCGTGGT-3′ and 5′-GGAGGATGCTGTTGTCTCGG-3′. The primers for *occludin* were as follows: 5′-CAGGTGCACCCTCCAGATTG-3′ and 5′-TGGACTTTCAA-GAGGCCTGG-3′. The primers for *claudin-1* were as follows: 5′-CTGCTTCTCTCTGCCTTCTG-3′ and 5′-GGAAGGCGAAGGTTTTGGAT-3′.

The expression level of NF-κB in the colon tissue was measured by the ELISA kit (Shanghai Ruifan Biotechnology, China), according to the instructions of the manufacturer.

### 2.6. Measurement of inflammatory factors

On day 16, nine mice were randomly selected from each treatment group (three mice per replicate), blood was collected from the eyeballs, and serum IL-6 was determined with the Mouse IL-6 ELISA kit (Boster Biological Technology, China). IL-1β was determined with the Mouse IL-1β ELISA kit (Shanghai Enzyme-linked Biotechnology, China), while TNF-α was determined with the Mouse TNF-α ELISA kit (Shanghai Yuanmu Biotechnology, China). LPS concentration was detected by the Mouse LPS ELISA kit from Shanghai Shuhua Biotechnology Co., Ltd. The protocols were followed according to the instructions of the manufacturer.

### 2.7. Microbiota analysis

The gut microbiota was analyzed as in our previous research (Li et al., 2021). The DNeasy PowerSoil kit (QIAGEN, United States) was used to extract the microbial genomic DNA from the cecum contents of mice. A NanoDrop ND-1000 spectrophotometer (Thermo Fisher Scientific, USA) quantified the DNA, and the quality was estimated using 0.8% agarose gel electrophoresis. Using high-quality DNA samples as templates, the bacterial 16S rRNA gene was amplified. The target fragments were extracted using an Axygen gel recovery kit (SelectScience, UK) and quantified on a microplate reader (BioTek, USA). TruSeq Nano DNA LT Library Prep kit (Illumina, USA) was used to construct the library, and the sequences were compared and analyzed using MOTHUR software and sequence clustering software Usearch (version 7.1). Greengenes is used as a comparison database. The data were deposited in NCBI SRA under the accession number PRJNA836642.

### 2.8. Statistical analysis

Data were analyzed using ANOVA in SPSS 22.0, and multiple comparisons of means were performed using Duncan's test.

## 3. Results

### 3.1. HAs treatment alleviates DSS-induced colitis in mice

At the end of the 16 days experiment, the body weight of the mice in the DSS_HA group was significantly higher (*p* = 0.047) than that of the mice in the DSS group and was not significantly different from that of the mice in the CTRL group (Figure 1C), suggesting that HAs have a positive effect on growth restriction caused by diarrhea.

**Figure 2:**
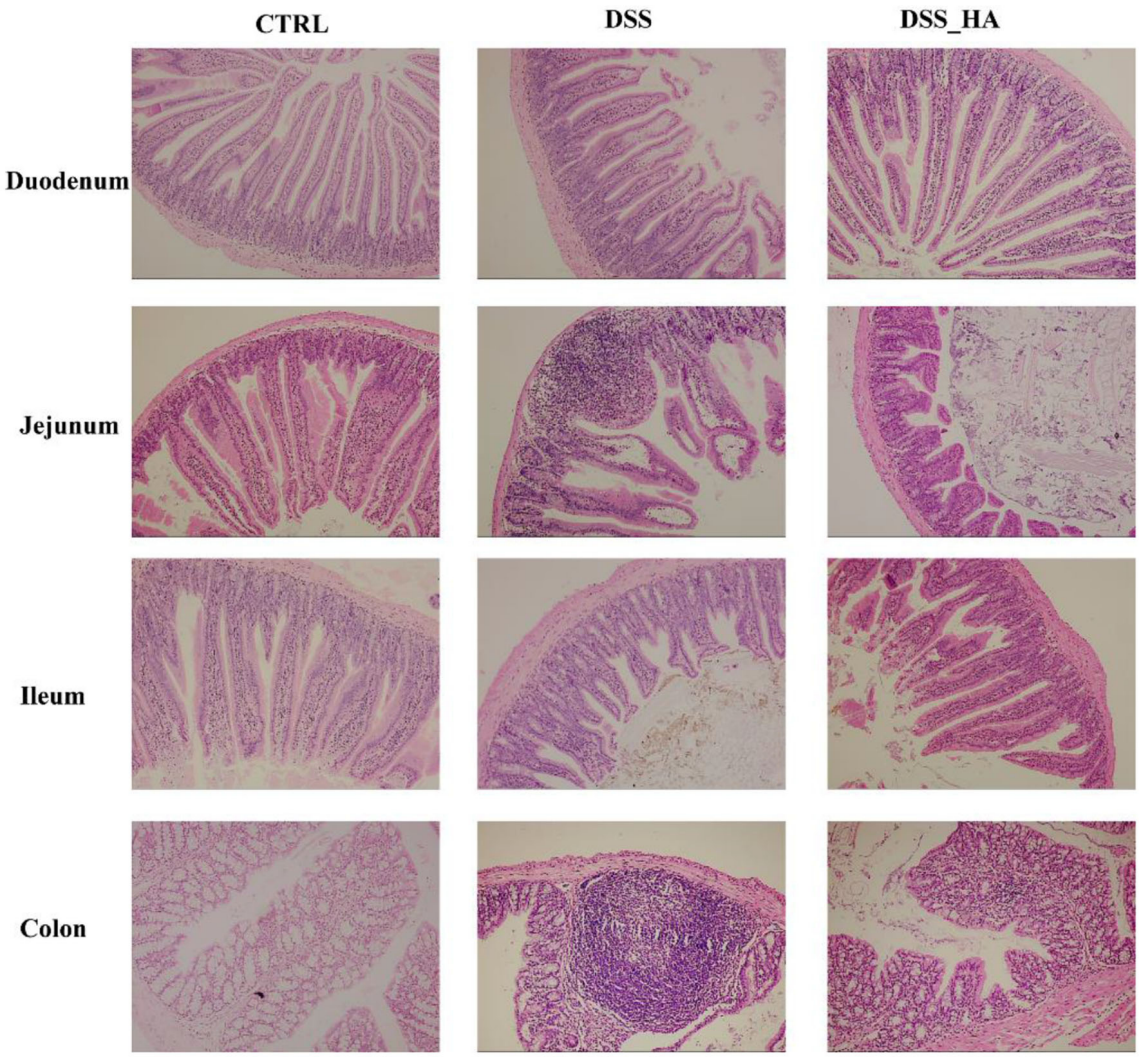
Hematoxylin and eosin staining of the duodenum, jejunum, ileum, and colon in the CTRL, DSS, and DSS_HA groups.

We performed H&E staining to visualize the intestinal tissue (Figure 2). In the DSS group, crypts in the duodenum were damaged, and inflammatory cells infiltrated the duodenum. In addition, the muscle layer was thinner than that in the CTRL group, and the submucosa was remarkably peeled off in the duodenum. In contrast, the structure of the crypts in the DSS_HA group was similar to that in the CTRL group, suggesting that HAs can prevent and repair crypt damage caused by DSS. In the jejunum of the DSS group, remarkable inflammatory cell infiltration was detected in the crypts with the disappearance of the crypt structure at the inflammatory site. The muscle layer was thinner than that in the control group. However, the degree of inflammatory cell infiltration was reduced in the DSS_HA group with the muscle layer restored. In the ileum, slight submucosal dissection was observed in the DSS group. However, the crypts were deformed, and the shape of the intestinal villi was severely damaged in the DSS_HA group, even though the shape of the intestinal villi was restored to a certain extent. Finally, the mice in the DSS group showed remarkable inflammatory cell infiltration in the colon compared with those in the control group, and the mucosal goblet cells were damaged. In contrast, inflammatory cell infiltration had disappeared in the DSS_HAs group, and the goblet cells were repaired to a certain extent. These results suggest that HAs can decrease the degree of inflammation in the intestinal tract, repair the shape of the intestinal villi and crypts, and ameliorate colitis in mice.

### 3.2. HAs treatment alters the composition of gut microbiota in mice

**Figure 3:**
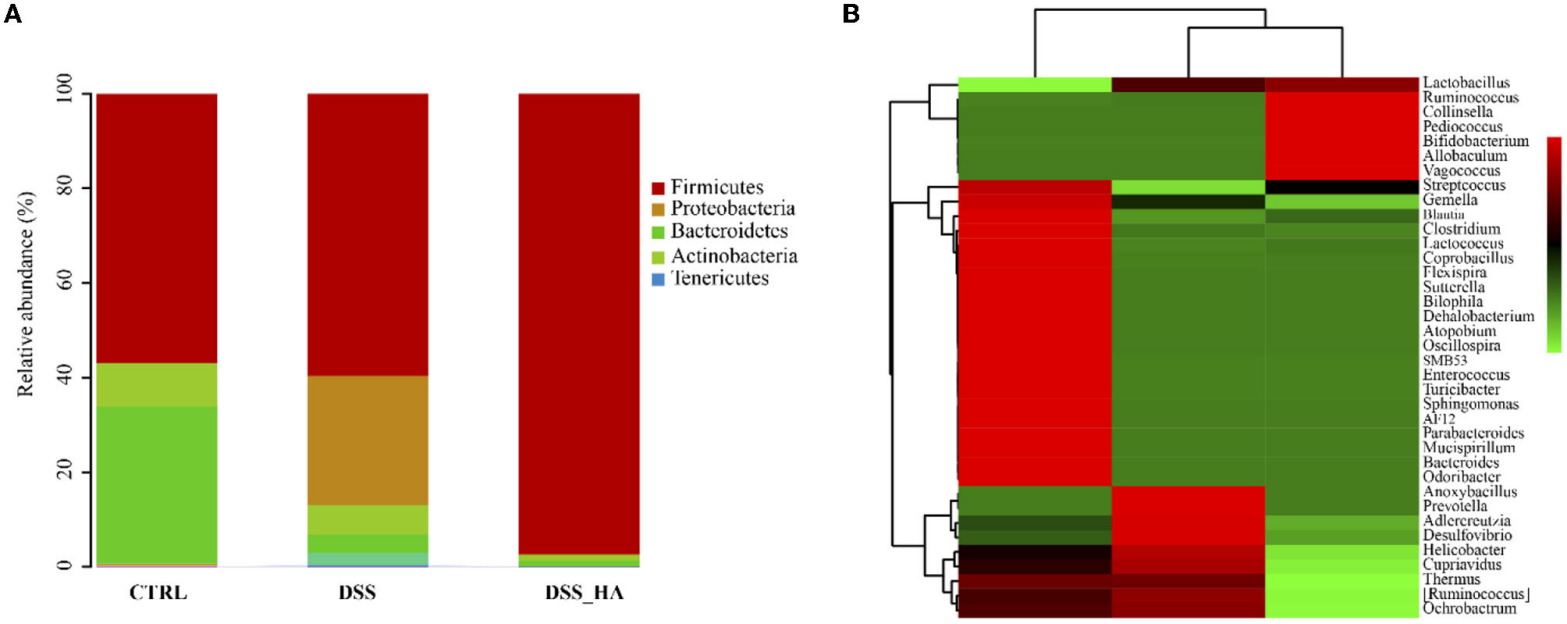
HAs alter the bacterial community of the gut in mice. **(A)** Taxonomic composition of the microbiota in the CTRL, DSS, and DSS_HA groups at the phylum level. **(B)** Heatmap of the top 50 dominant genera in each group.

After feeding on HAs, Proteobacteria decreased substantially and *Firmicutes* increased abundantly in the DSS_HA group (Figure 3A). To understand this alteration in detail, the top 50 abundant bacteria were analyzed at the genus level (Figure 3B). The results showed that *Streptococcus, Gemella, Blautia, Clostridium*, and other genera increased in the DSS group compared with the bacteria in the CTRL group. In contrast, *Lactobacillus, Ruminococcus, Collinsella, Pediococcus*, and *Bifidobacterium* increased substantially in the DSS_HA group compared to those in the CTRL and DSS groups. This implies that HAs increased the abundance of profitable bacteria, such as *Lactobacillus* and *Bifidobacterium*.

### 3.3. HAs treatment alleviates colitis through the Toll-like receptor 4-NF-κB pathway

**Figure 4:**
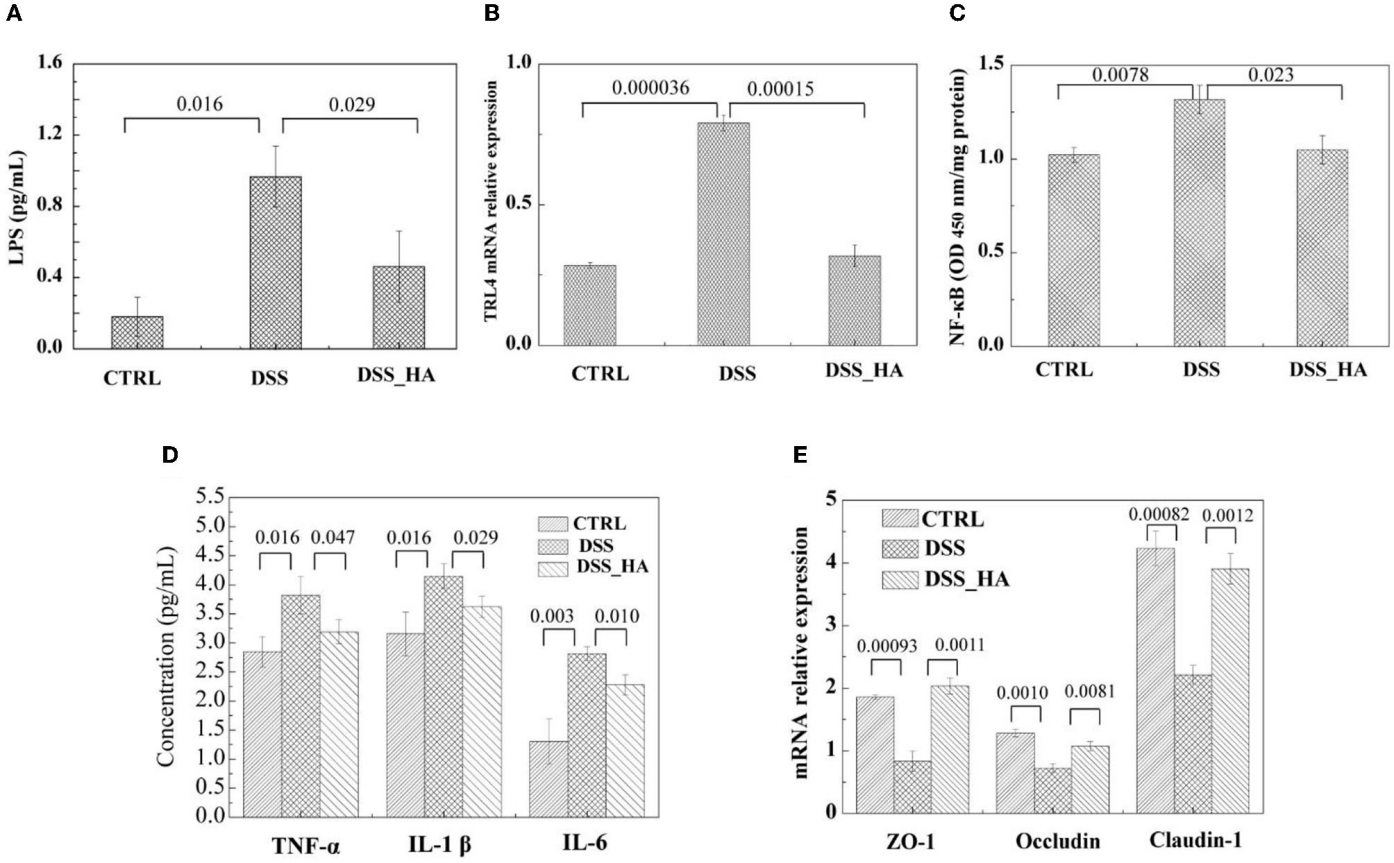
HAs protected mice from inflammatory bowel disease induced by DSS exposure. **(A)** Lipopolysaccharide (LPS) concentration in the serum of the mice in each group. **(B)** Relative Toll-like receptor 4 (TLR4) expression level in the colon tissue of each group. **(C)** NF-κB level in the colon tissue of each group. **(D)** Comparison of the TNF-α, IL-1β, and IL-6 concentrations in the mice sera of the three groups. **(E)** Relative expression level of *ZO-1, occludin*, and *claudin-1* in the colon of the three groups. The *p*-values from **(A–E)** are shown at the top of the histogram.

Higher concentrations of LPS were detected in the DSS group, whereas treatment with HAs significantly decreased the LPS concentration (*p* = 0.029) (Figure 4A). It has been reported that LPS could stimulate the TLR4-NF-κB signaling pathway to induce the release of TNF-α, IL-1β, and IL-6, which are inflammatory cytokines in inflammatory bowel disease. When we analyzed the expression of TLR4 and cytokine concentrations, the results indicated that DSS could induce the expression of TLR4 and NF-κB, whereas HAs could inhibit this expression (Figures 4B, C). In addition, the serum concentrations of the three cytokines were significantly higher in the DSS group than in the control group (*p* = 0.016, 0.016, and 0.003, respectively) (Figure 4D). However, TNF-α, IL-6, and IL-1β concentrations were significantly lower in the DSS_HA group than in the DSS group (*p* = 0.047, 0.029, and 0.010, respectively). These results are consistent with those of morphological analysis performed using intestinal HE staining, suggesting that HAs can be used to alleviate inflammation.

Hence, we analyzed the effect of DSS and HAs on the expression of intestinal *occludin, ZO-1*, and *claudin-1*, which are critical tight junction proteins. The results showed that DSS decreased the expression of *ZO-1, occludin*, and *claudin-1*. In contrast, the mRNA levels of the three genes increased in the DSS-HA group (Figure 4E), suggesting that HAs are beneficial for maintaining the integrity of the intestinal barrier.

## 4. Discussion

Dextran sulfate sodium (DSS) can induce intestinal inflammation by damaging the epithelial monolayer and disseminating the proinflammatory intestinal contents into underlying tissue. The DSS colitis model is widely accepted and popular in inflammatory bowel disease (IBD) research (Chen et al., 2022; Peng et al., 2022).

We investigated whether HAs could alter the gut microbiota in mice. At the phylum level, adding DSS to the diet markedly increased Proteobacteria in the gut microbiota of the group compared with the CTRL group, which is similar to the previous study (Zhu et al., 2021; Deng et al., 2022; Li W. et al., 2022). The alteration of the gut microbiota motivated us to study the effect of the gut microbiota on mice. Proteus produces lipopolysaccharide (LPS), a pro-inflammatory toxin that induces intestinal inflammation in mice easily (d'Hennezel et al., 2017; Jeong et al., 2019; Jia et al., 2022; Zhong et al., 2022). The results showed that HAs could counteract LPS-induced inflammation, which might be related to the reduction of the relative abundance of Proteobacteria by HAs.

It has been reported that LPS could stimulate the TLR4-NF-κB signaling pathway to induce the release of TNF-α, IL-1β, and IL-6, which are inflammatory cytokines in inflammatory bowel disease (Gerges et al., 2020). This is consistent with our experimental results. We further investigated the potential mechanism of the function of HAs. In healthy mice, normal TLR4-NF-κB signal transduction is an important basic immune mechanism for maintaining the integrity of the epithelial barrier and the tolerance of the microbiota. However, in inflammatory bowel disease, TLR4-NF-κB overexpression and overactive TLR4-NF-κB signal transduction may damage intestinal mucosal homeostasis, leading to tissue damage and inflammation (Cario, 2010). Previous study has found that in DSS-induced colitis, TLR4-NF-κB are overexpressed (Hou et al., 2013). This is consistent with our experimental results. Compared to the DSS group, HAs significantly reduced the relative expression levels of TLR4 and NF-κB.

Alteration of the gut microbiota is related to intestinal permeability (Kang et al., 2022; Koutoukidis et al., 2022; Zhang et al., 2022). The intestinal barrier is key to blocking toxic substances and maintaining normal intestinal homeostasis. Previous studies have shown that DSS-induced colitis is associated with the disruption of intestinal barrier function (Gerges et al., 2020). In this study, HAs can reduce the degree of intestinal inflammation, repair the shape of the intestinal villi and crypts, and improve colitis in mice. At the same time, HAs increased the relative expression of intestinal tight junction protein mRNA. Therefore, we believe that HAs can regulate intestinal barrier function and prevent toxic substances from leaking from the intestine into the circulatory system.

**Figure 5:**
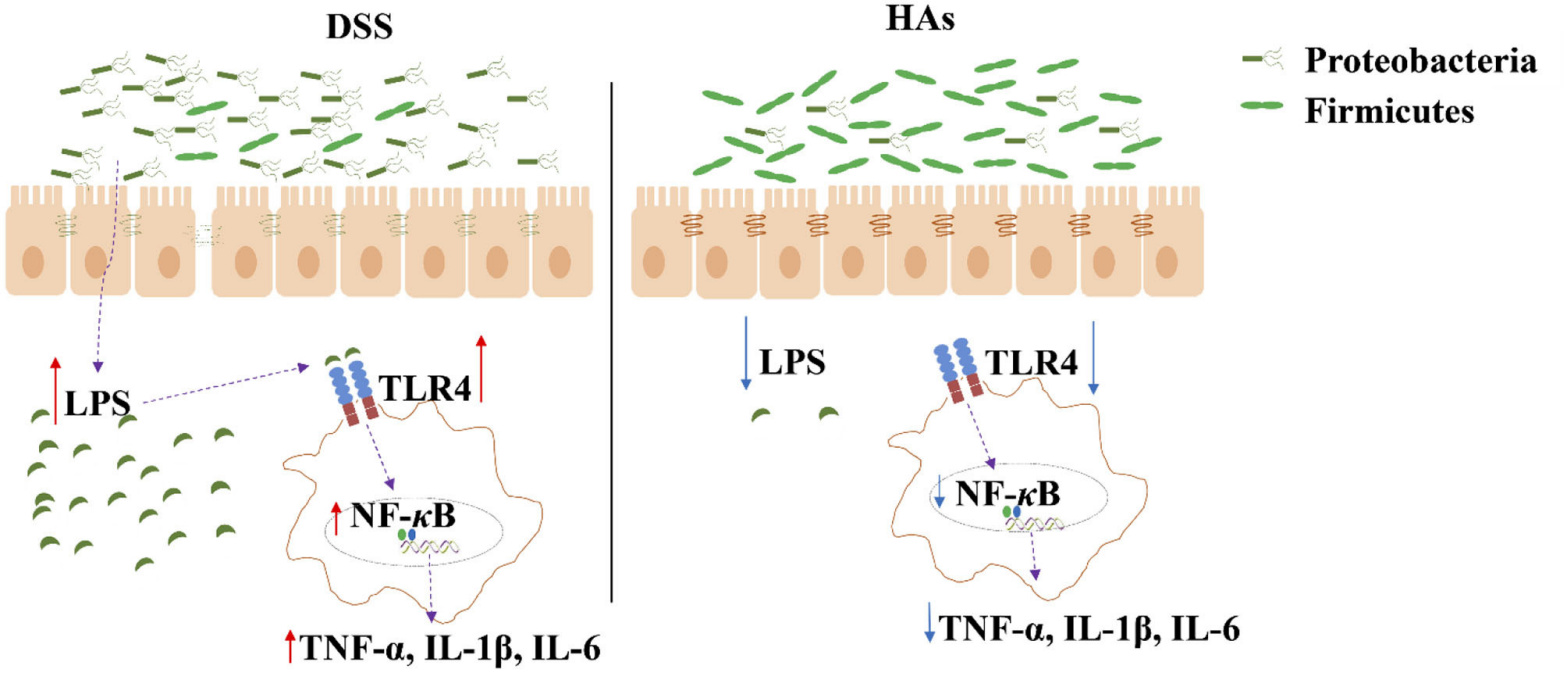
Illustration of the mechanism by which HAs reduce inflammation. HAs decrease the abundance of Proteobacteria but increase that of *Firmicutes*. The reduced level of Proteobacteria decreases LPS concentration, which further inactivates the expression of TLR4 and the production of inflammatory cytokines (TNF-α, IL-1β, and IL-6).

In conclusion (Figure 5), we showed that DSS might have caused colitis in mice by altering the intestinal morphology, whereas HAs restored the morphology and decreased the levels of inflammation. Furthermore, we observed that HAs can alter the community of the gut microbiota damaged by DSS. By increasing the abundance of *Firmicutes* and decreasing the abundance of Proteobacteria, HAs decreased the expression of TLR4 and further reduced the levels of inflammatory cytokines. HAs also increased intestinal permeability, which was damaged by DSS. These results suggest that HAs have the potential to treat colitis. Despite the valuable findings of our study, it is important to acknowledge its limitations. One major limitation is that the exact mechanism by which HAs alleviate colitis remains unknown. However, our research revealed that the addition of HAs to the diet of aging laying hens led to significant alterations in the alpha and beta diversities of the intestinal microbiome (Li C. et al., 2022). Specifically, we observed an increase in the levels of short-chain fatty acids, including acetic acid, isovaleric acid, and isobutyric acid, in the HA group. These results suggest that the primary benefit of HAs in treating colitis may be through the modulation of the intestinal microbiota and the alteration of microbial metabolites, further reducing inflammation, maintaining mucosal barriers, and inhibiting pathogen growth. Further research is required to elucidate the detailed functions and mechanisms of HAs in the treatment of colitis.

## Data availability statement

The datasets presented in this study can be found in online repositories. The names of the repository/repositories and accession number(s) can be found below: PRJNA836642.

## Ethics statement

The animal study was reviewed and approved by the Animal Care and Use Committee of the Qilu University of Technology (Shandong Academy of Sciences).

## Author contributions

PX, JH, and JZ designed the research. MS, BW, and CZ performed the experiments and analyzed the data. All authors reviewed the results and approved the final version of the manuscript.
